# Neuroinformatics challenges to the structural, connectomic, functional and electrophysiological multimodal imaging of human traumatic brain injury

**DOI:** 10.3389/fninf.2014.00019

**Published:** 2014-02-26

**Authors:** S. Y. Matthew Goh, Andrei Irimia, Carinna M. Torgerson, John D. Van Horn

**Affiliations:** Department of Neurology, Institute for Neuroimaging and Informatics, Keck School of Medicine, University of Southern CaliforniaLos Angeles, CA, USA

**Keywords:** neuroinformatics, traumatic brain injury, neuroanatomy, connectomics, rehabilitation, MRI, DTI

## Abstract

Throughout the past few decades, the ability to treat and rehabilitate traumatic brain injury (TBI) patients has become critically reliant upon the use of neuroimaging to acquire adequate knowledge of injury-related effects upon brain function and recovery. As a result, the need for TBI neuroimaging analysis methods has increased in recent years due to the recognition that spatiotemporal computational analyses of TBI evolution are useful for capturing the effects of TBI dynamics. At the same time, however, the advent of such methods has brought about the need to analyze, manage, and integrate TBI neuroimaging data using informatically inspired approaches which can take full advantage of their large dimensionality and informational complexity. Given this perspective, we here discuss the neuroinformatics challenges for TBI neuroimaging analysis in the context of structural, connectivity, and functional paradigms. Within each of these, the availability of a wide range of neuroimaging modalities can be leveraged to fully understand the heterogeneity of TBI pathology; consequently, large-scale computer hardware resources and next-generation processing software are often required for efficient data storage, management, and analysis of TBI neuroimaging data. However, each of these paradigms poses challenges in the context of informatics such that the ability to address them is critical for augmenting current capabilities to perform neuroimaging analysis of TBI and to improve therapeutic efficacy.

## INTRODUCTION

Traumatic brain injury (TBI) affects ~1.7 million people in the United States every year, leading to roughly 50,000 cases of mortality and 80,000 cases of permanent severe neurological disability annually (Ghajar, 2000; Faul et al., 2010). Throughout the past few decades, the use of neuroimaging to acquire knowledge of injury-related effects upon brain function and recovery has become prominent due to the recognition that spatiotemporal computational analyses of TBI evolution are useful for capturing the effects of its dynamics (Irimia et al., 2011). On the other hand, the proliferation of neuroimaging studies has brought about the need to analyze, manage, and integrate TBI neuroimaging data with sophisticated neuroinformatics methods which can address and handle their large dimensionality and informational complexity.

The high dimensionality of TBI neuroimaging data poses one of the most significant challenges to the development and implementation of data processing workflows for TBI analysis. This dimensionality stems partly from the fact that various types of magnetic resonance imaging (MRI) sequences reveal only certain aspects of TBI pathology, which implies that their combined use is often necessary in order to acquire a comprehensive view of TBI lesion type and extent. For instance, fluid attenuated inversion recovery (FLAIR) and susceptibility weighted imaging (SWI) are MRI sequence types which are suitable for detecting edema and cerebral micro-hemorrhages, respectively (Irimia et al., 2011). Partly because of such qualitative and quantitative differences between MRI sequence types as well as between MRI and other imaging modalities such as computed tomography (CT) and positron emission tomography (PET), a vital component of TBI neuroimaging involves the availability of multimodal neuroimaging data sets to aid in the identification and characterization of pathology.

Present methodologies for long-term clinical assessment of this condition include the use of scoring scales such as the Glasgow Coma Scale (GCS), which is a frequently used evaluator of consciousness level and head injury severity. Additional clinical measures of functional outcome after TBI which are used in clinical practice include acute physiology and chronic health evaluation (APACHE), Mortality Probability Model (MPM), and simplified acute physiology score (SAPS; Vincent and Moreno, 2010), all of which can be complemented by neuroimaging-based metrics. In the case of the GCS and of other currently available scoring systems, their effectiveness in providing prognostic information is hampered by their limited descriptiveness. By contrast, computational analyses of multimodal structural neuroimaging data offer a variety of ways in which pathological changes can be assessed. It is important to note that (a) the GCS is typically used in conjunction with a number of other clinical measures and physiological metrics, and that (b) computational analyses vs. clinical scoring systems fulfill different roles. Thus, one theme of this review is that the drawbacks of conventional clinical scoring systems can be complemented by outcome prediction models formulated using neuroinformatics tools, which require the exploration and mining of quantitative metrics derived from structural neuroimaging data.

Given the current trends in TBI neuroimaging, this review aims to highlight and draw the attention of the neuroinformatics community to the challenges encountered in the study of human TBI within the context of three distinct types of neuroimaging: structural, connectivity, and functional. It attempts to suggest how novel data-driven solutions should be formulated to assist TBI neuroimaging analysis with the ultimate purpose of improving therapeutic efficacy. The analytic approaches examined below outline the use of a varied number of neuroimaging techniques and demonstrate the wealth of knowledge obtainable through quantitative analysis of neuroimaging data. We propose that, to improve rehabilitation strategies and the accuracy of TBI patient outcome prediction, it is necessary to augment existing capabilities to facilitate the multimodal use of neuroimaging methods and of their application to large population samples of TBI patients, as well as to individual patients by means of personalized approaches. This task should be reliant on continued development, support and input from neurologists, neuroinformaticians and biostatisticians to provide the theoretical tools and practical mechanisms required for technological and scientific progress in this field of high priority to public health.

## STRUCTURAL NEUROIMAGING APPROACHES

Computational methods for the analysis of brain structure provide a powerful approach to the investigation of TBI-related pathology. Typical quantitative metrics for the study of brain structure include morphometric measures (e.g., the curvature and folding index of the cortex) and volumetric measures – e.g., cortical thickness, gray matter (GM) volume, white matter (WM) volume, etc. – which have been highly useful in describing neuroanatomical profiles at the macroscopic level, in both health and in a variety of pathological conditions (Ashburner and Friston, 2000; Thompson et al., 2003). One motivating factor behind the decision to undertake the calculations of these metrics within large collaborative efforts such as the Alzheimer’s Disease (AD) Neuroimaging Initiative (ADNI) has been the desire to identify biomarkers which are prognostic and informative of clinical outcome, and which can be used to optimize the formulation of patient treatment as well as the selection of rehabilitation protocols, as in the study of Jack et al. (2008). The latter authors aimed to address the neuroinformatics challenge of longitudinal ADNI data processing by (a) linking all data at each time point, (b) making a repository available to the scientific community, (c) developing technical standards for longitudinal imaging studies, (d) determining optimum methods for image acquisition and analysis, and (e) validating imaging biomarker data. Such goals are excellently suited for future human TBI studies as well. All of these tasks involve neuroinformatics approaches which are currently insufficiently available in human TBI research. Subsequently, the ability to perform relevant systematic and quantitative analyses of TBI brain structure has been appreciably affected by a number of formidable challenges which this section aims to highlight.

One challenge encountered during the task of constructing TBI data analysis workflows for the extraction of clinically relevant information is the task of tissue segmentation, a process often associated with the three-dimensional analysis of MRI volumes. In neuroimaging, tissue segmentation refers to the classification of voxels from MRI data into relevant tissue types (e.g., GM, WM, cerebrospinal fluid, non-cortical structures) so that morphometric and volumetric measures can be quantified. Typically, tissue segmentation is a complex procedure involving the correction of magnetic field inhomogeneities, image intensity normalization, extra-cerebral voxel removal via skull-stripping, and the assignment of each voxel to one of several classes (WM, GM, etc.) using a probabilistic model based on image intensity differences between voxels belonging to each class (Dale et al., 1999). Though there are a wide variety of approaches to segmentation including those based on machine learning (Powell et al., 2008; Hofmann et al., 2011), brain tissue segmentation often incorporates the application of anatomical priors while computing the probability of a voxel belonging to a certain tissue type (Irimia et al., 2011). Whereas the application of such anatomical priors is typically quite feasible in the case of healthy brains, this class of methods is known to fail when applied to moderate or severe TBI volumes because, in such cases, (a) TBI neuroanatomy can differ substantially from health due to the presence of gross pathology and (b) edema and hemorrhage can dramatically alter voxel intensities, thereby modifying the spatial mapping of such voxels to atlas space in an undesirable manner. Thus, segmentation of TBI volumes can be particularly difficult to automate due to the heterogeneity of injury location, shape, and size, none of which are easily predictable (Filippi et al., 1998). Nevertheless, it is important to acknowledge that neuroimaging analysis of mild TBI exhibiting no gross pathology can typically be accommodated using standard algorithms, although for moderate and/or severe TBI more sophisticated methods are needed, as previously stated. Given the fact that most automatic segmentation algorithms have been developed for healthy brains or for brains with diminutive amounts of gross pathology (Irimia et al., 2012c), implementing such algorithms for moderate to severe TBI cases often necessitate periodic user intervention and guidance. This suggests that future data-processing workflows devised for facilitating TBI segmentation should aim to accommodate and minimize the need for periodic user intervention. Presently, a persistent challenge resides in the methodological dichotomy of opting for either a manual or automatic segmentation approach. While manual segmentation methods do not require (complex) segmentation algorithms, such methods are significantly more costly than automatic ones due to the comparably large amount of time and human resources needed for adequate segmentation of even a single MRI volume. Furthermore, the nature of manual delineation implies that substantial inter- and intra-observer variability are to be expected, which may increase quantitative measurement errors and thereby diminish the statistical power of inferential tests applied to sets of such measurements (Kempton et al., 2011). In the case of TBI lesions, however, one benefit of manual segmentation is that it is often more trustworthy than conventional automatic segmentation algorithms which were developed for the tissue classification of healthy brains (Lehmann et al., 2010), largely because TBI pathology is extremely heterogeneous among subjects. On the other hand, conventional automatic segmentation algorithms greatly reduce data processing time and improve reproducibility, but can suffer from appreciable inaccuracies in the case of TBI.

One software package which is often used for automatic segmentation and whose methodological capabilities are illustrative of automatic segmentation packages in general is FreeSurfer (Dale et al., 1999). As in the case of typical unsupervised segmentation packages, FreeSurfer has been thoroughly validated in healthy brains and in some diseases exhibiting structural pathology types which are more moderate and more predictable than those encountered in TBI (Du et al., 2007; Jovicich et al., 2009). Nevertheless, automatic tissue classification algorithms including FreeSurfer remain imperfect and can experience inaccuracies in skull stripping, WM/GM boundary identification, etc. even in healthy subjects (Strangman et al., 2010). Any such defects may require user input to (a) add control points and thereby aid FreeSurfer to identify WM, (b) remove unlabeled voxels representing the dura mater and thereby correct skull stripping, and/or (c) manually restore WM/GM portions which had been inappropriately removed during first-pass segmentation. In addition, typical automatic segmentation methods do not perform lesion classification, which suggests that additional user-guided segmentation is needed in this step as well.

**FIGURE 1:**
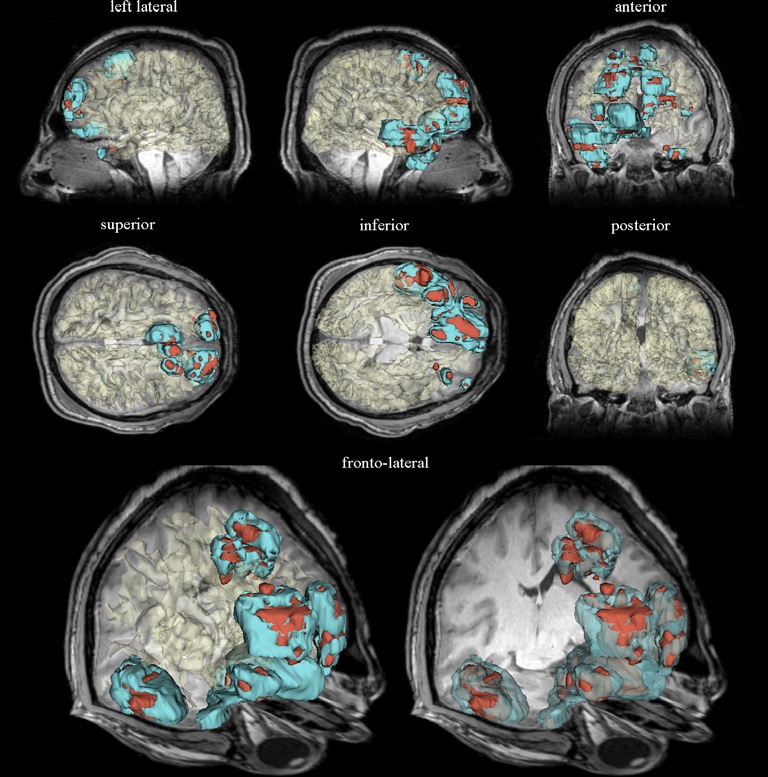
**Three-dimensional models of semi-automatically segmented healthy-appearing and pathology-affected tissues are displayed for a sample patient with severe TBI within a neuroinformatics framework.** Representative slices of the *T*_1_ volume acquired 3 days after injury are superimposed. Models of edematous and hemorrhagic tissues are colored in cyan and dark red, respectively. The WM surface was segmented automatically using FreeSurfer, demonstrating the capabilities of this software package to perform automatic tissue classification of healthy-appearing tissues. The WM model is translucent in each brain view to facilitate the visibility of anatomic details obviated in the MR volume slice displayed. See Irimia et al. (2011) for a detailed description of the neuroinformatics methodology used to generate these visualizations.

In comparison to conventional tissue classification methods, a number of sophisticated segmentation methods now exist which have adopted more sophisticated approaches to address the task of TBI tissue segmentation. As stated, TBI pathology is often gross and highly heterogeneous, even in comparison to other types of neuropathology such as AD. In some cases, pathology patterns may present image intensities and appearance similar to those of normal tissues (Irimia et al., 2012c), necessitating segmentation algorithms tailored to analyzing pathology. In dealing with the similar problem of MR volume segmentation in multiple sclerosis (MS), Van Leemput et al. (2001) proposed a method which detects MS lesions as outliers with respect to a statistical model for the healthy brain, rather than attempting to model such lesions explicitly. The model interleaves (a) statistical classification of the image voxels into a number of healthy tissue types, (b) evaluation of whether each voxel truly belongs to healthy tissue, and (c) estimation of intensity distribution parameters and MR bias field parameters only based on healthy tissue voxels. Voxels not well constrained by the statistical model for normal brain MR images are detected as voxels containing MS lesions. Another sophisticated approach designed specifically for TBI (Irimia et al., 2012c; Wang et al., 2012) employs multimodal neuroimaging data from multiple time points to improve segmentations and to describe changes in healthy tissue and pathology. Their framework utilizes several semi-automatic segmentation tools available within 3D Slicer, a freely available software environment for image processing where automatic segmentation can be complemented by additional user evaluation (Irimia et al., 2011). Examples of semi-automatic segmentations obtained using such workflows are shown in **Figure 1**. Similar algorithms have been derived from approaches for the MR analysis of brain sclerosis and tumors, which present problems similar to those of TBI lesion segmentation (Prastawa et al., 2003, 2004). Other algorithms such as the one developed by Wu et al. (2006) use multimodal MRI to classify MS lesions into several subtypes, each of which can be analyzed to represent different outcome measurements.

Finally, because standard registration and segmentation methods do not account for changes in image appearance across time, sophisticated methods have been developed to jointly estimate a space deformation and a change in image appearance which can lead to the construction of a spatiotemporal trajectory which smoothly transforms the structural volume acquired from the patient at one time point into the volume acquired at a subsequent time point. In particular, algorithms such as that of Niethammer et al. (2011) have the ability to explain changes in image appearance by (a) a global deformation, (b) a deformation within a geometric model, and (c) an image composition model. The development of such longitudinal registration methods is motivated by the challenge to predict long-term effects of TBI based on longitudinal changes in tissue types and in their spatial configuration, which may provide further clinical insight into the prediction of tissue fate and patient outcome.

The wealth of MR segmentation algorithms is an indication that segmentation, at least in the case of TBI, is a complicated task which can be solved through many approaches. However, this wealth, arguably, is also an indication that no single approach has been demonstrably superior. Many of these methods, in fact, still require user intervention and post processing. Therefore, automatic segmentation may be an appropriate problem for the neuroinformatics community to address by means of data mining and novel workflow designs.

## CONNECTIVITY NEUROIMAGING APPROACHES

As discussed in the previous section, conventional structural neuroimaging methods enable the calculations of volumetrics and morphometrics, which can reveal important information on gross anatomy changes effected by brain injury upon the brain in general and upon cortical structures in particular. By contrast, the advent of modern neuroimaging methods which allow the observation of neuronal circuitry *in vivo *(such as diffusion tensor imaging, DTI) has perpetuated the interest in connectivity mapping, and further allows investigation of connectivity changes in brain injury patients. The benefit of DTI in contrast to dissection and to WM staining is that the former can be used noninvasively in human patients, which is a major advantage in human studies. Techniques such as DTI tractography enable the mapping of macroscopic WM connections, which can yield descriptive metrics of brain connectivity, including fiber bundle length and connectivity density (Wang et al., 2012).

The ability of DTI tractography methods to reconstruct area-to-area connectivity in TBI has been the topic of multiple validation studies (Mori and van Zijl, 2002; Dauguet et al., 2007; MacDonald et al., 2007; Skudlarski et al., 2008), including one study by the present authors, where area-to-area connectivity counts obtained via DTI using purpose-built software were independently validated by three researchers with experience in neuroanatomy (Van Horn et al., 2012). Whereas DTI is certainly not as accurate for reconstructing area-to-area connectivity as some invasive methods (e.g., *post-mortem* dissection and WM staining), its ability to capture connectivity information accurately has been found to be quite reasonable provided that the size of each brain parcel denoting a graph node is sufficiently large compared to the DTI voxel size (Irimia et al., 2011; Irimia et al., 2012c; Van Horn et al., 2012).

It has been acknowledged (Meythaler et al., 2001) that 40–50% of TBI patients exhibit diffuse axonal injury (DAI), a mechanism of brain injury which is microscopic in nature such that conventional CT and MRI are typically insufficient to capture it in detail. DTI, on the other hand, is more ideally suited to non-invasively measure the diffusion of molecules through biological tissue. Whereas diffusion of water along healthy axons is predominantly anisotropic, studies using DTI have indicated that DAI may be detected as a reduction in diffusion anisotropy (Arfanakis et al., 2002). With the advancement of such techniques, the goal of characterizing TBI-related changes in brain connectivity can be pursued by using brain water diffusion data to reconstruct WM tracts three-dimensionally, to visualize fiber cluster integrity and to locate gross anatomy changes prompted by injury.

To study WM changes prompted by TBI, neuroimaging researchers have adopted various mathematical approaches to aid in data analysis, the most prominent of these being network theory. This approach typically focuses upon the task of reconstructing brain networks using graphs, which are mathematical representations consisting of nodes (vertices) and links (edges) between pairs of nodes. Such representations have long been used to represent brain networks (Strogatz, 2001), though their popularity for the purpose of systematic connectivity mapping in humans via noninvasive techniques such as DTI has only increased appreciably throughout the past decade (Bullmore and Sporns, 2009; Rubinov and Sporns, 2010). In the context of neural connectivity, nodes represent brain regions which exhibit some given functional or anatomical pattern. Links, on the other hand, denote the presence or absence of connections, and can be weighted to represent the strengths of neural connections between distinct areas (Strogatz, 2001; Rubinov and Sporns, 2010). The manner in which nodes and links are defined can vary substantially, depending on the set of conventions used to parcellate the brain. In many cases, parcellation schemes are used to delineate gyri and sulci into homogenous regions which correspond to graph nodes (Thirion et al., 2010; Stanley et al., 2013). The advantage of this approach is that each graph node corresponds to an anatomical region whose identity and spatial extent have been well documented by neuroanatomists (Irimia et al., 2012c; Van Horn et al., 2012).

The application of network theory within TBI neuroinformatics has increased in recent years (Achard et al., 2012; Irimia et al., 2012c; Van Horn et al., 2012; Wang et al., 2012), with a special focus upon identifying network patterns which can offer insight into the long-term effects of TBI. A study by Pandit et al. (2013), for example, utilizes the tools of network theory to investigate changes in brain network topology following TBI, to the effect that the victims of this condition exhibit abnormalities with respect to normal controls from the standpoint of several global network-theoretic measures, including total connectivity, average path length and network efficiency. Thus, one advantage of DTI which is highly beneficial to the study of TBI is the fact that this imaging modality allows the extraction of network-theoretic connectivity information from which patient-specific measures can be computed, including metrics of centrality, assortativity, node degree, etc. (Achard et al., 2012; Irimia et al., 2012a). Statistical comparison of such measures between TBI patients and healthy control subjects can outline the nature, extent and location of TBI damage upon neural pathways, and may also reveal information which can be useful when formulating personalized rehabilitation strategies.

Network metrics can be used to investigate patterns of connectivity changes in TBI patients and to inform clinicians who wish to incorporate the use of this knowledge into the process of treatment formulation. This trend is already under way in the study of other disorders of the nervous system; for example, previous studies have found significant differences in network-theoretic metrics (e.g., spatial pairwise clustering and intra-nodal homogeneity) when comparing healthy adults to schizophrenics (Zalesky et al., 2012), AD patients, and to normal aging. Thus, the informatics relevant to these studies offers new ways to quantitatively characterize changes in anatomical network patterns, including the means to relate WM network topology to brain function. These techniques are particularly relevant in TBI due to the well-known facts that (a) brain injury can cause dramatic changes in WM connectivity (Kinnunen et al., 2011; Irimia et al., 2012a) and that (b) such changes often result in the deterioration of cognitive function (McDowell et al., 1997; Chen and D’Esposito, 2010). Because cognitive deficits incurred as a result of injury may either ameliorate or deteriorate over time depending on a variety of factors (Hoofien et al., 2001; Kraus et al., 2007), neuroinformatics approaches designed for professionals in the field of TBI (e.g., TBI clinicians, epidemiologists, public health professionals, etc.) are well-suited for providing clinicians and researchers with advanced tools for investigating the temporal evolution of TBI WM lesion profiles. This may lead to an improvement of current understanding on how neurological damage leads to functional impairment, and may also spur the development of pathology-tolerant neuroimage analysis tools which can be applied to other types of brain injury, such as stroke and MS.

Despite the widespread application of diffusion imaging over the years, several fundamental technical challenges remain only partially resolved. One persistent difficulty has been the challenge of correcting for head movement in the MR scanner. Head motion not only interferes with image acquisition, but may also lead to errors in the calculation of diffusion tensor scalars such as fractional anisotropy (FA) and mean diffusivity (MD), as shown in a number of studies (Ling et al., 2012; Van Dijk et al., 2012). It should be noted that head motion is not unique to connectivity neuroimaging and that it is also a concern in structural neuroimaging. Approaches to mitigating head motion in non-head injury patients have included the use of anesthesia (Karlik et al., 1988; Holshouser et al., 1993), which is often used when neuroimaging data are acquired from acute injury patients in a neurointensive care setting. Naturally, however, this approach may not be suitable in all TBI cases, and therefore the integration of motion correction algorithms into post-processing steps remains critical to the usability of the acquired data. Investigators have systematically examined the residual effects of head motion in diffusion imaging, and have reported the impact of head motion upon the calculation of diffusion metrics. Tijssen et al. (2009) found a positive bias between head motion and FA in regions with low anisotropy; in regions with higher anisotropy, head motion was found by these authors to artifactually decrease FA. Ling et al. (2012) reproduced these findings and expanded on the findings of Tijssen et al. by examining the residual effects of motion following conventional motion correction frameworks (i.e., image registration, gradient table adjustment, diffusion weighted image removal). This is especially problematic in TBI studies where diffusion metrics may incorrectly represent the presence or absence of pathology-affected tissue. Thus, further research into the development of effective motion correction algorithms is particularly critical in the context of TBI research.

Another challenge resides in the somewhat limited ability of tracking algorithms to correctly infer the continuity of fibers from voxel to voxel. One drawback of probabilistic tractography which can affect TBI studies with predilection is that the latter is more likely to reconstructs short fibers, which can increase the probability that WM located near GM or near a lesion is assigned an inappropriately large number of tracts (Kuceyeski et al., 2011). Connectivity assessment may further be complicated by the presence of edematous or hemorrhaging tissue, where the appreciable isotropy of water diffusion interferes with the ability of DTI to capture fiber directionality. Yet another factor which TBI neuroinformatics tools should aim to account for is the difficulty of detecting crossing fiber bundles, particularly in peri-lesional regions. This phenomenon, which is traditionally known to be caused by limitations in current approaches for reconstructing fiber trajectories (Iturria-Medina et al., 2007; Bullmore and Sporns, 2009), can be particularly challenging to account for in TBI where changes in anisotropy are often prompted within and surrounding lesion sites.

A final concern for TBI connectivity analysis is the increasing need for versatile data visualization tools. While a large number of these exist, many of them, as Margulies et al. (2013) point out, are limited by the necessity to compromise and prioritize the representation of information in terms of anatomic vs. connectomic, aesthetics vs. informational content, and thoroughness vs. readability. For example, although *TrackVis* is intended for whole brain tractography visualization, its strength is primarily in data visualization rather than data processing and computation. By comparison, *OpenWalnut *is more tailored towards data processing due to the modularity of its software environment design and pipelining engine. However, while both of these tools fulfill the need for anatomic visualization, very few workflows exist which offer comprehensive summaries (i.e., anatomy, function, connectivity) of the human connectome as reconstructed via neuroimaging.

**FIGURE 2:**
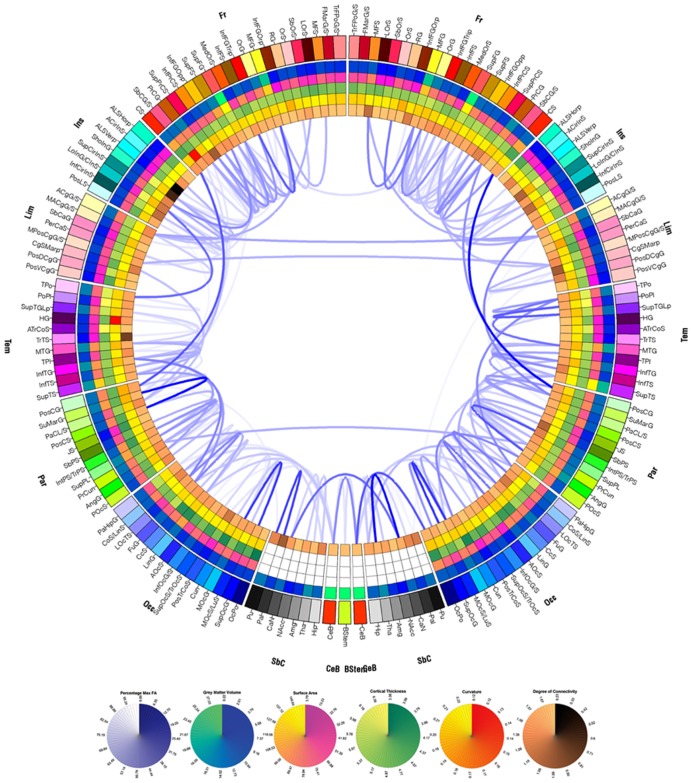
**Circular connectogram representation graphically displays WM atrophy over a 6 month period.** The left and right halves of the connectogram correspond to the left and right hemispheres, respectively. Each hemisphere of the brain is divided into frontal, insular, limbic, temporal, parietal and occipital lobes, as well as into subcortical structures, cerebellum, and the brain stem; the latter three are represented at the bottom of the circle. Each lobe is further divided into parcels (gyri and sulci in the case of the cortex) and is assigned a unique identifying color. Radially aligned, concentric rings represented using various color schemes depict various attributes of each corresponding brain parcel. From the outermost to the innermost one, the rings contain wedges which encode GM volume, surface area, cortical thickness, curvature, and degree of connectivity. A link of variable opacity is drawn between certain pairs of brain parcels, reflecting structural connectivity properties between regions. In the case of the connectogram displayed, links displayed indicate connections which suffered from large atrophy from the acute baseline to the chronic follow-up time point. Link transparency encodes the percentage change Δ in fiber density, in the range [min(Δ), max(Δ)], with larger changes (more negative values of Δ) being encoded by more opaque hues of blue. The lowest color opacity corresponds to the smallest absolute value of the percentage change which is greater than the selected threshold of 30%, and the highest opacity corresponds to the maximum absolute value of the change in fiber density. See Irimia et al. (2012a) for details.

Research on mapping and visualizing cortical connections has a relatively long history, beginning with animal model studies. Scannell and Young, in particular, have performed extensive work on the cat cerebral cortex in representing neural connectivity using a variety of graph depiction strategies (Scannell and Young, 1993; Scannell et al., 1995). Irimia et al. (2012a) have developed a graphical approach for representing TBI connectivity alterations, illustrating the location and extent of WM change over time in TBI patients. This visualization paradigm generates connectivity representations called “connectograms” using an informatically driven software package which allows brain connectivity information to be depicted within a circle of radially aligned elements. A connectogram from a sample TBI patient created using this approach is shown in **Figure 2**. The purpose of this figure is to illustrate the presence of appreciable atrophy due to TBI. Each circular wedge element represents a specific cortical region and is positioned on either side of the vertical axis, corresponding to the left or right hemisphere, respectively. The location of each fiber extremity is associated with the appropriate cortical parcellation of a sulcus or gyrus. Inter-region connectivity is represented by a link of variable opacity drawn the between radially aligned elements, and depends on fiber density as well as upon pathology severity. This mode of representation emphasizes the presence of atrophy, which is substantially more severe in TBI than in healthy aging, particularly over a 6-month period. For this reason, in contrast to **Figure 2**, it is to be expected that the connectogram displaying longitudinal changes in connectivity for a healthy adult would reveal considerably fewer and weaker changes over a 6-month period, particularly for a young or middle-aged adult.

The connectogram as a graphical representation method offers a succinct means of displaying longitudinal differences in WM connections and highlights the current impetus for incorporating neuroinformatics approaches into the development of brain connectivity visualization methods (Margulies et al., 2013). Advances in robust connectivity visualization and representation methods could encourage longitudinal studies, which depend on neuroinformatically driven workflows to process the large amounts of data associated with capturing and quantifying connectivity changes across multiple time points. Armed with measurements of morphologic and connectomic alterations over time, customized publication database search strings may additionally be crafted and submitted to PubMed or Google Scholar to return literature relevant to damage in the affected areas, the effects on connectivity, and putative treatment options (Irimia et al., 2012a). Recent approaches to information retrieval, extraction and analysis of the neuroimaging literature, such as those of Bug et al. (2008) and Keator et al. (2013) may provide additional starting points for the development of flexible tools for the description and retrieval of neuroscience-relevant resources, as pioneered by the Neuroscience Information Framework (NIF).

## FUNCTIONAL IMAGING AND NEUROPHYSIOLOGICAL APPROACHES

Functional neuroimaging modalities and electrophysiological recordings allow researchers to investigate behavioral deficits as well as the pathophysiological responses of the brain following injury. The techniques most frequently employed include functional MRI (fMRI), electroencephalography (EEG), magnetoencephalography (MEG), and PET. Each of these techniques possesses varying levels of applicability with inherent strengths and weaknesses depending on the aims of the study, as well as on the condition of the patient. Accordingly, it would be beneficial to develop data mining, processing and analysis approaches which can facilitate the optimization of information usage acquired across various functional imaging modalities.

Whereas fMRI is useful in post-injury investigations of cerebral activation patterns during the performance of cognitive tasks, its reliability in diagnostic applications may be impeded by factors such as increased intracranial pressure, which can alter hemodynamic responses and, subsequently, its measure of cerebral activity (Hillary et al., 2002). In such cases, the use of EEG may be preferable to that of fMRI or PET due to the high temporal resolution of the former (in the millisecond range), and to the fact that EEG does not rely on indirect measures of activity such as the hemodynamic response. Nevertheless, it is useful to note that the temporal resolution gap between fMRI and EEG may be partially alleviated through the use of novel multi-band methods for fMRI, which involve shorter acquisition times and thus greater temporal resolution (Moeller et al., 2010; Ugurbil, 2012). One limitation of EEG to consider, however, is the fact that the structural changes and presence of pathology prompted by TBI may increase the difficulty of localizing pathophysiological activity recorded after acute brain injury. Specifically, electrical source localization is a problematic task due to the ill-posed nature of the bioelectric inverse problem. The latter refers to the task of localizing the sources of brain activity based on scalp EEG measurements. By contrast, the calculation of electric potentials produced at the scalp due to current sources in the brain is known as the forward problem of bioelectricity (Lima et al., 2006; Irimia et al., 2013a). Additionally, appreciable cancellation of cortical signals occurs in EEG (Goh et al., 2013; Irimia et al., 2013a,b). Accurate localization of cortical activity depends on a number of factors, one of which is the anatomic faithfulness of the head model used in the forward calculation of electric potentials (Gencer and Acar, 2004; Goh et al., 2013). EEG localization studies involving models which account for the presence of lesions and cavities have shown that the latter can have significant qualitative and quantitative effects upon the computed electric potentials (He et al., 1987). Thus, from an informatics standpoint, it is necessary to develop data processing tools which incorporate realistic head model generation and which can account not only for head anatomy and tissue conductivity profiles, but also for the effects of tissue conductivity changes due to TBI. Comparatively, MEG presents advantages which are unique and often complementary to EEG. For example, a single head volume model is typically sufficient in MEG forward modeling, partly because the biomagnetic fields of the brain are far more dependent on tissue permeability rather than conductivity. Whereas conductivity can vary considerably across biological tissues, their permeability is always very nearly equal to that of free space (*μ*_0_), such that the use of a single head volume is justified. An immediate consequence of this fact is that, whereas the spatial distribution of electric potentials over the scalp is smeared and attenuated due to the high resistivity of the skull, magnetic field recordings are nowhere near as strongly affected by the conductivity profile of the head, which is advantageous in MEG experiments (Lima et al., 2006; Sharon et al., 2009; Irimia et al., 2012b). In addition, the number of sensors used for MEG recordings (e.g., 306 sensors in the Elekta Neuromag^®^ MEG scanner) is often higher than that of EEG montages, where fewer than 256 sensors are typically used. Finally, MEG sensors can usually sample brain signals at higher frequencies and signal-to-noise ratios than EEG electrodes. Nonetheless, MEG scanners are available only at a handful of brain research centers, and data acquisition costs for this modality are prohibitively higher than for EEG. Future data-processing tools devised for acquiring and analyzing brain signals from TBI patients should aim to be user-friendly, regardless of whether EEG or MEG is used. In this context, the requirement of user friendliness implies that the approaches for data acquisition and analysis should be intuitive to grasp and easy to use by clinicians and by other health professionals who are unfamiliar with the complexities of anatomical modeling and of inverse localization methods for EEG-based neurophysiological signal analysis.

**FIGURE 3:**
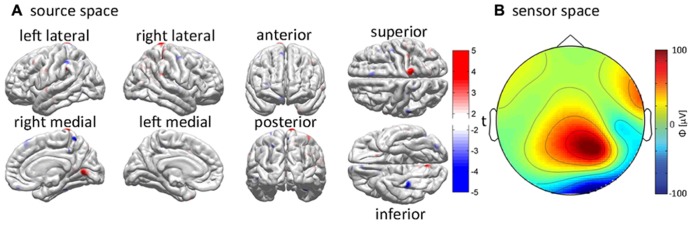
**Example of EEG inverse localization in a sample acute TBI patient using an integrative pipeline.** The cortical sources responsible for the generation of recorded EEG waveforms are determined using the application of a minimum norm inverse localization method. **(A)** EEG potentials recorded over the scalp (i.e., in “sensor space”) are inversely localized onto the cortical surface (i.e., into “source space”). The inverse estimate of the cortical activity responsible for the generation of EEG signals is plotted using *t *scores, which indicate the likelihood for each cortical location to be electrically active. The magnitude of *t *indicates whether the localized electric current is oriented out of (*t* > 0, red hues) or into (*t* < 0, blue hues) the cortex. **(B)** The interpolated values of the potentials measured at each sensor location are mapped over an idealized, circular representation of the scalp to generate a topographic map. Color indicates the magnitude of the recorded electric potential ϕ in μV. See Irimia et al. (2013b) for further details.

Although a variety of functional neuroimaging and electrophysiological techniques can and have been used in neurotrauma research, a large number of functional TBI studies are uni-modal in the sense that they employ only a single technique to obtain quantitative values of a specific measure. Naturally, it would be more advantageous to combine multiple modalities in order to achieve a more comprehensive view of how brain injury leads to subsequent functional losses. An insufficient number of studies have accomplished this, however, due to the difficulty associated with integrating data acquired across various measurement modalities. Research involving the localization of brain activity after TBI using EEG includes three recent studies (Goh et al., 2013; Irimia et al., 2013a,b) where the combined use of MRI and EEG is demonstrated. In both of these studies, cortical electrical activity is inversely mapped over the cortex with clinical applications to the localization of epileptogenic foci in post-traumatic epilepsy (PTE). An example of this approach is shown in **Figure 3**. In these studies, the effects of pathology upon forward modeling and inverse source localization were explored in the context of a semi-automatic, multimodal neuroimaging approach involving anatomically faithful TBI head models containing 25 tissues types, including six types accounting for TBI-related pathology. The multimodal aspects of these studies highlight the combined use of structural and functional imaging data using an inverse localization algorithm subject to anatomic constraints provided by MRI.

In a general sense, neuroimaging-based methodologies have not yet addressed the paucity of strategies for integrating multivariate connectivity data with other imaging modalities including fMRI, PET, EEG, and MEG. The ability to extract meaningful information from multimodal data must often make use of dimensionality reduction techniques, as well as multivariate statistical inference methods which can allow researchers to test statistical hypotheses based on large descriptive feature vectors. One study which illustrates the integration of functional neuroimaging modalities to the benefit of TBI research is by Storti et al. (2012), who integrated fMRI and EEG to evaluate PTE in patients with pharmacologically resistant epilepsy. During MRI scanning, the patients who participated in this study were additionally equipped with an MR-compatible EEG amplifier and cap arranged in the 10/20 montage. The combined use of these modalities allowed the authors to compare clinical semiology, BOLD activation, and source localization which could only be obtained as a result of the advantages offered by the complementary nature of combined fMRI/EEG. As previously stated, conventional fMRI alone offers high spatial resolution, but poor temporal resolution, whereas EEG alone offers high temporal resolution but relatively poor spatial resolution in the absence of inverse localization. Multimodal neuroimaging is ideally suited for TBI clinical care because different modalities can reveal distinct information about injury. For example, an MRI FLAIR sequence can reveal the presence and spatial extent of brain edema, whereas an SWI sequence is ideally suited for the detection of microhemorrages. Thus, the fusion of such multimodal information can provide substantial insight into the structural profiles of lesions, thereby helping to formulate clinical interventions. Nevertheless, despite the trend toward integration of modalities to study TBI across all its stages, it has been proposed that the use of fMRI and PET is more appropriate during the sub-acute to chronic stages, as opposed to the acute phase where the presence of increased intracranial pressure is likely and may lead to misleading measurements (Hillary et al., 2002). In chronic TBI, by contrast, metrics of brain function derived from fMRI and PET have been used by various researchers to investigate neuropsychiatric performance (Kasahara et al., 2011; Palacios et al., 2013).

The motivation for diversifying the range of functional neuroimaging modalities which are typically included in analyses of brain structure has increased considerably as neuroimaging analysis methods have become more sophisticated. In this respect, one key point to address in functional TBI neuroimaging studies is the fact that large volumes of data are often generated in the course of neuroimage acquisition and analysis. Specifically, data acquired using modalities such as fMRI, EEG and MEG incorporate a time dimension: (a) in the case of multiband fMRI, the additional 3D nature of this modality can make data storage a very substantial challenge; (b) in the cases of EEG and MEG, the high temporal resolution (in the MHz range, though typically down-sampled to the kHz range or lower) can also raise storage-related challenges. Collectively, these properties of functional neuroimaging data can result in substantial storage demands from dedicated databases and repositories (Van Horn and Toga, 2009). An examination of fMRI articles from representative issues of the journal *Neuroimage* found that since 1995, the amount of data collected has doubled approximately every 26 months (Van Horn and Toga, 2009, 2013). At this rate, it is projected that data storage requirements may exceed 20 GB per published study by the year 2015. Consequently, it is vital that funding agencies should support the computational infrastructure needed to accommodate multimodal data, and that hardware resource availability should develop alongside at the same pace. Next-generation neuroinformatics approaches to the management of multimodal data should also be developed, particularly for the purpose of inter-institutional collaborations and data sharing.

An important recent trend in the consideration of functional TBI neuroimaging has been the proliferation of approaches involving data-intensive discovery – rather than hypothesis testing – in TBI research (Akil et al., 2011). The net result of this trend has been the need for centralized databases to assist the research community in terms of hardware infrastructure and efficiency of data mining. Whereas a number of neuroimaging databases exist which are dedicated to the gathering and dissemination of neuroimaging data for various types of diseases including ADNI (Jack et al., 2008; Jack et al., 2010; Weiner et al., 2012), such large-scale database systems are only now becoming available for the purpose of TBI neuroimaging research, including the informatics system of the Federal Interagency Traumatic Brain Injury Research (FITBIR, fitbir.nih.gov). In addition to FITBIR, the NIF (www.neuroinfo.org) is another useful resource established to survey and compile a list of neuroscience databases, tools, and materials so that researchers can efficiently search across a variety of smaller, individual databases.

For FITBIR, NIF and other resources and databases dedicated to the task of disseminating data and functional neuroimaging analysis software to the research community, one challenge which requires careful consideration is the need for data sharing and storage mechanisms to accommodate large collaborations across multiple research centers with wide geographic distributions. The intrinsic necessity for multidimensionality in TBI neuroimaging data sets entails the reality that inter-institutional TBI research may require hardware data storage capabilities in excess of those needed by other large neuroimaging collaborative efforts such as ADNI, for example, which does not need to rely as heavily as TBI research does upon data multimodality. Furthermore, it would be highly beneficial for researchers to benefit from neuroinformatics-driven data sharing capabilities which can facilitate collaborations among researchers from various institutions as well as among clinical and research staff responsible for acquiring TBI neuroimaging data (Manley and Maas, 2013).

## DISCUSSION

Despite the emerging trend towards the use of multimodal imaging by TBI experts, the capacity to acquire and process large amounts of neuroimaging data remains dependent upon the availability of sophisticated imaging hardware and large-scale computational resources to store and manage such data. Additionally, extracting meaningful and clinically useful information from multimodal neuroimaging data can necessitate advanced neuroimaging processing software packages which are capable of handling their multi-dimensionality and inherent complexity. Although improvement of TBI treatment and rehabilitation protocols by means of multimodal neuroimaging remains a critical goal to healthcare providers, much of the ability to accomplish this aim is dependent upon the identification of clinical biomarkers which are predictive of TBI pathology progression, and the future of TBI neuroinformatics must therefore accommodate the use of statistical prediction models which aid in forecasting TBI clinical outcome.

Computational neuroanatomy can aid TBI outcome prediction by providing quantitative metrics for further analysis rather than by resorting to the task of discerning voxel intensity differences visually or to similar types of qualitative observations. By definition, quantitative structural imaging studies utilize mathematical computations which can be reliably reproduced and applied across entire cohorts, and such undertakings can be facilitated through the use of neuroinformatics. Nevertheless, when considering the task of performing inferential statistical analyses of neuroimaging-derived structural metrics in TBI, it is also critical to incorporate statistical techniques which can accommodate and account for the attrition rates encountered in longitudinal studies of this condition. Specifically, one-third to one-half of TBI study participants are lost to follow-up primarily due to low socioeconomic status, substance abuse history, and violent injury etiology (Corrigan et al., 2003). This can be detrimental to the validity of outcome studies, and data processing workflows tailored for structural neuroimaging analyses should therefore implement biostatistical techniques for addressing the problem of missing measurement data in order to account for the attrition rates encountered in longitudinal studies of this population.

The wealth of information which can be extracted from connectivity analyses has spurred the development of graph-theoretic quantitative approaches to describe brain network organization following TBI. The methodologies of classical graph theory have lent their power to the study of complex networks such as those in the brain, and the resulting approaches have been beneficial to the task of quantifying the networks of the brain with high reliability and reproducibility using a manageable number of neurobiologically meaningful and easily computable quantitative measures (Rubinov and Sporns, 2010). Furthermore, network-theoretic metrics can be robust to the use of distinct cortical parcellations across studies as well as to various approaches for quantifying functional connectivity. This is particularly useful in the case of TBI because investigating relationships between brain structure, neurological damage, and functional impairment is essential when attempting to formulate patient-specific rehabilitation protocols.

The goals of numerous TBI neuroimaging studies can be greatly facilitated by the use of neuroinformatics protocols to streamline and perform data analysis, but the availability solutions to facilitate the study of brain structure, function and connectivity remains insufficient. This is partly due to the intricate complexities of the human brain and its functions, and partly due to the fact that neuroimaging-based methodologies for its study have not yet fully matured. Structural, connectomic, and functional data are highly multidimensional, which frequently demands the use of sophisticated statistical methods for multivariate analysis. Current data processing efforts for their joint analysis continue to be hampered by the need for considerable manual customization steps which are often needed to bridge compatibility gaps between the various software environments employed. For instance, to perform anatomically faithful forward/inverse calculations in EEG, head model generation requires not only the segmentation of healthy-appearing tissues – which can be performed more or less automatically – but also the segmentation of pathology-affected tissues, which is often performed manually, as outlined in the first section. However, because little compatibility typically exists across software environments and the algorithms used for each of these processing steps, neuroinformatically informed strategies are necessary to invoke the integration of neuroimage segmentation tools with forward model generation modules, inverse localization algorithms, and other methodologies for the analysis of brain functional data.

In conclusion, next-generation TBI neuroinformatics must address the need to develop integrative workflows which (a) perform automatic tissue segmentation of TBI pathology, (b) lead to a reduction in the number of algorithmic approaches and software environments required for connectomic and functional analysis, (c) minimize the amount of time and effort devoted by the user to manual intervention, and which (d) promote knowledge extraction leading to targeted clinical intervention. Such integration can allow researchers to generate strategies for analyzing brain function after injury, for extracting clinically useful information from each modality, for combining information obtained from each modality, and for gaining insight into the relationships between brain metabolism, cerebral blood flow, and cortical electrical activity underlying successful recovery in TBI.

## Conflict of Interest Statement

The authors declare that the research was conducted in the absence of any commercial or financial relationships that could be construed as a potential conflict of interest.
